# Economic and social values in the brain: evidence from lesions to the human ventromedial prefrontal cortex

**DOI:** 10.3389/fneur.2023.1198262

**Published:** 2023-10-12

**Authors:** Despina Messimeris, Richard Levy, Raphaël Le Bouc

**Affiliations:** ^1^FrontLab, Paris Brain Institute (ICM), Sorbonne University, INSERM UMRS 1127, CNRS UMR 7225, Pitié-Salpêtrière Hospital, Paris, France; ^2^Department of Neurology, Pitié-Salpêtrière Hospital, Sorbonne University, Assistance Publique-Hôpitaux de Paris (AP-HP), Paris, France; ^3^Motivation, Brain and Behavior Laboratory (MBB), Paris Brain Institute (ICM), Sorbonne University, INSERM UMRS 1127, CNRS UMR 7225, Pitié-Salpêtrière Hospital, Paris, France

**Keywords:** reward value, social value, ventromedial prefrontal cortex, orbitofrontal cortex, lesion studies, decision-making

## Abstract

Making good economic and social decisions is essential for individual and social welfare. Decades of research have provided compelling evidence that damage to the ventromedial prefrontal cortex (vmPFC) is associated with dramatic personality changes and impairments in economic and social decision-making. However, whether the vmPFC subserves a unified mechanism in the social and non-social domains remains unclear. When choosing between economic options, the vmPFC is thought to guide decision by encoding value signals that reflect the motivational relevance of the options on a common scale. A recent framework, the “extended common neural currency” hypothesis, suggests that the vmPFC may also assign values to social factors and principles, thereby guiding social decision-making. Although neural value signals have been observed in the vmPFC in both social and non-social studies, it is yet to be determined whether they have a causal influence on behavior or merely correlate with decision-making. In this review, we assess whether lesion studies of patients with vmPFC damage offer evidence for such a causal role of the vmPFC in shaping economic and social behavior.

## 1. Introduction

**Figure 1:**
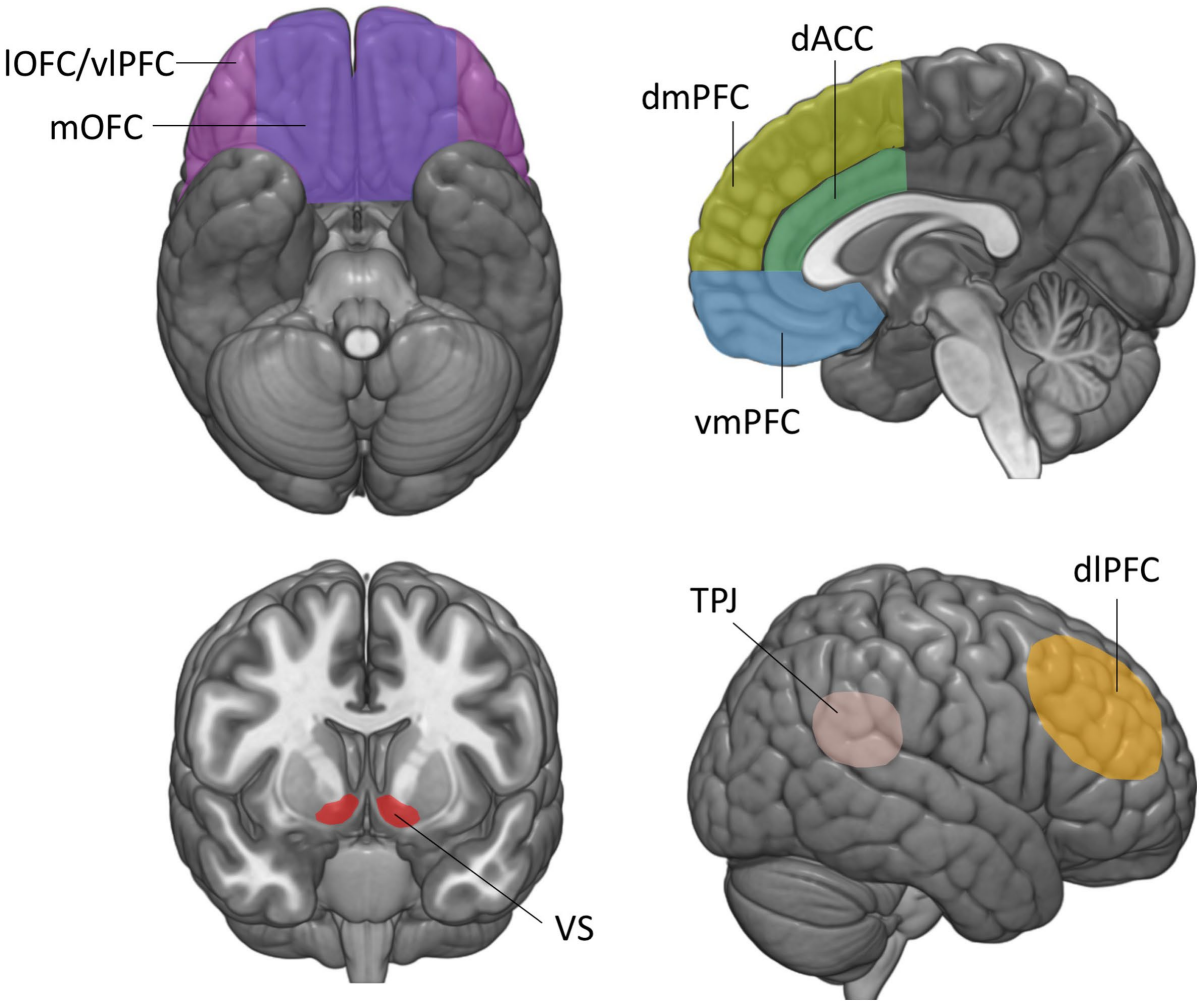
Brain regions involved in the construction of economic and social value. The vmPFC is defined here as the medial orbitofrontal cortex and lower portions of the medial prefrontal cortex and anterior cingulate cortex, below the genu of the corpus callosum. mOFC, medial orbitofrontal cortex; lOFC, lateral orbitofrontal cortex; vmPFC, ventromedial prefrontal cortex; vlPFC, ventrolateral prefrontal cortex; dmPFC, dorsomedial prefrontal cortex; dlPFC, dorsolateral prefrontal cortex; dACC, dorsal anterior cingulate cortex; *VS*, ventral striatum; TPJ, temporo-parietal junction.

Throughout the history of neurology, individual patient cases have played a major role in deepening our understanding of brain-behavior relationships. Among these cases, Phineas Gage and patient EVR, have generated enduring interest in the role of ventral areas of the prefrontal cortex, including the ventromedial prefrontal cortex (vmPFC, Figure 1). These two prototypical cases triggered a paradigm shift in neurology, from an era where the vmPFC was considered as a silent or less prominent cortex, to the view that the vmPFC plays a pivotal role in shaping human behavior. Following brain damage in ventromedial and orbital prefrontal areas, both patients displayed striking changes in personality and manner, despite seemingly preserved basic intellectual abilities (1–4). The story of these famous cases and their behavioral impairments have been documented in great details over the years. Here, we just briefly outline two aspects of the behavioral changes of these patients that appear relevant for the purpose of this review. First, both patients seemed impaired in value-based decision-making. Gage’s initial description stated that he “*does not estimate size or money accurately, though he has memory as perfect as ever. He would not take $1,000 for a few pebbles which he took from an ancient river bed where he was at work*” (4). Similarly, for EVR, “*deciding where to dine might take hours, as he discussed each restaurant’s seating plan, particulars of menu, atmosphere, and management. He would drive to each restaurant to see how busy it was, but even then, he could not finally decide which to choose. Purchasing small items required in-depth consideration of brands, prices, and the best method of purchase*” (2). Second, Gage and EVR also demonstrated impaired abilities in social decision-making. While Gage showed suspected impairments in social norm compliance and empathy, being described as “*irreverent, indulging at times in the grossest profanity (which was not previously his custom) … manifesting but little deference for his fellows*” (3), EVR showed poor judgment in choosing valuable social partners. For example, he began a short-lived relationship with a disreputable woman, and became “*involved in a home-building partnership with a former coworker, a man of questionable reputation who had been fired from the company. Despite warnings by family and friends, EVR invested all his savings in the partnership. The business failed and he had to declare bankruptcy, losing his entire investment*” (2).

Although Gage and EVR cases pointed to a fundamental role of the vmPFC in social and non-social decision-making, the precise nature of the function(s) supported by this region has been long debated. Several theories have been put forward to propose a unified function of the vmPFC, including the somatic marker (5) and the affective meaning hypotheses (6). More recently the “cognitive map” hypothesis postulated a role for the vmPFC in learning the structure of the world by representing the relationships between its different states (7–10). Another theoretical framework, the “extended common neural currency” hypothesis, suggests that identical neural processes in the vmPFC assign values, or the motivational relevance, to social and non-social factors (11). This framework builds on theories proposed in the field of non-social decision-making. Empirical studies in that field have identified a set of brain regions, including the vmPFC and the ventral striatum, as part of a brain valuation system. Neural activity in these regions is thought to encode a “common neural currency,” allowing the value of different rewards or actions to be compared on a common scale, which is essential for guiding decision-making across different contexts (11). Recent observations showing the involvement of the brain valuation system during social decision-making have suggested extending the “common neural currency” hypothesis to social factors (11). In that schema, identical neurons in the vmPFC would process social and non-social value signals, although they may incorporate inputs from different brain regions that process information relevant for social or non-social contexts (11). By contrast, a “social valuation specific schema” assumes the existence of a distinct population of neurons, that may or may not be located in the vmPFC, dedicated specifically to computing values in social contexts (11).

Note that functional imaging studies implicating the vmPFC in reward valuation (12, 13) provide correlational, not causal, evidence for its role in value processing. Demonstrating the causal role of the vmPFC with non-invasive brain stimulation techniques is difficult as current methods mostly target brain areas at the cortical surface. Human lesion studies provide an alternative method to demonstrate the critical role of damaged regions.

Here, we aimed at assessing whether human vmPFC lesion studies support a causal role for the vmPFC in both social and non-social decision-making. We define social decisions as choice situations involving more than one person. We chose to focus this review on published works that could address the “extended common neural currency” hypothesis and establish whether the vmPFC represents the values of both social and non-social factors. We searched for vmPFC lesion studies spanning from 1990 to 2023 by querying the MEDLINE database with the following terms: (‘vmPFC’ OR ‘ventromedial prefrontal’ OR ‘orbitofrontal’ OR “medial prefrontal’) AND (‘patients’ OR ‘lesion’ OR ‘damage’). We only included group studies that used social or value-based decision-making tasks in patients with focal lesions in the vmPFC. We excluded clinical case studies, studies involving only patients with non-focal lesions such as neurodegenerative diseases or non-penetrating traumatic brain injuries, and studies that did not employ behavioral tasks addressing either social or non-social decision-making (for reviews on the effect of vmPFC damage on all cognitive domains, see (14, 15)).

This review is structured as follows. First, we provide an overview of lesion studies implicating the vmPFC in representing the economic value of various goods during non-social decision-making. We then review lesion studies that have examined the role of the vmPFC in assigning values to the diverse contexts and interactions during social decision-making. For both economic and social values, we start off by summarizing key results from functional imaging studies identifying value signals in the vmPFC, and then outline the impairments in decision-making that result from vmPFC damage. Note that the functional imaging studies presented here are intended to provide a summary of the main findings related to each decision-making task, not to provide a comprehensive review on brain activations in social and non-social decision-making, which would fall beyond the scope of this review. We group the presentation of lesion studies by the type of valuation process or social situation they address. Note that in this review, we refer to the vmPFC as the medial orbitofrontal cortex (OFC) (Figure 1, area 14) and lower portions of the medial prefrontal (mPFC) and anterior cingulate cortex (ACC) below the genu of the corpus callosum (Figure 1, areas 24, 25, 32) (16), since imaging and lesion studies hardly dissociated these regions. Finally, we discuss open questions that could contribute to a more comprehensive understanding of the function of the vmPFC in shaping social and non-social behavior.

## 2. Economic values in the vmPFC

Dysfunction of the vmPFC has been associated early with abnormal goal-directed behaviors, including apathy, lack of initiative, poor judgment, indecisiveness and defective decision-making in particular in the economic domain. Later work led to the hypothesis that the vmPFC might represent the economic value of different goods in a common neural currency. In this section, we refer to economic values as the motivational and hedonic relevance of non-social options, such as material goods, monetary rewards, food etc. Economic value signals have been examined in the brain at three distinct stages of the decision-making process: during the receipt and consumption of rewards, when learning from obtained rewards, and when choosing rewards. These different stages involve distinct types of value signals: experienced value, anticipated value and decision value. We therefore group the presentation of studies on economic values according to these three types of neural value processes.

### 2.1. Experienced values

Experienced values signals correspond to the neural activity associated with the immediate hedonic aspects of receiving or consuming a reward, for example the outcome of a choice. Such value signals have been found in a set of brain regions that constitute the brain valuation system, including the ventral striatum (*VS*) and the vmPFC. In the vmPFC, neural activity has been associated, for example, with the subjective pleasantness of receiving monetary rewards, different types of food (e.g., snacks, juices, milkshakes, wine, etc.), or various goods (e.g., trinkets, pieces of music, movies, etc.) (13, 17–20). A causal role of the vmPFC in hedonic responses would therefore predict reduced expression of pleasure, i.e., anhedonia, after vmPFC damage. Few studies, however, have examined this prediction. Damage to the vmPFC was found to reduce self-reported happiness compared to prefrontal lesions outside of the vmPFC (21). However, in a larger population of combat veterans with penetrating brain injuries, bilateral lesion to the vmPFC was not associated with higher anhedonia in self-reported scales and clinical interviews (22). Moreover, in a behavioral gambling task, patients with vmPFC damage showed preserved pleasantness ratings and emotional autonomic responses when experiencing monetary gains (23, 24). Although these patients experienced weaker disappointment and no regret in the task (i.e., the effect on hedonic experience of unobtained outcomes and unchosen gambles, respectively), further studies suggested that those impairments reflected ventrolateral rather that ventromedial damage (24). Lesion studies therefore provide little evidence for a causal role of the vmPFC in hedonic experience. This is consistent with the idea that the vmPFC is involved in “*coding”* but not in “*causing”* pleasure (25), in the sense that it encodes a signal that correlates with hedonic experience without underlying this mental experience. However, such a signal that scales with the hedonic experience of reward might constitute a prerequisite to learn to anticipate future rewards and later guide decision-making.

### 2.2. Anticipated values

Anticipated values signals reflect the prediction of the experienced value associated with the different options under consideration. These signals have been observed in functional imaging studies in the *VS*, the OFC and the vmPFC (26–29). During value learning, anticipated value is thought to be dynamically updated based on “reward prediction error” (RPE) signals. Prediction errors measure deviations from individuals’ reward expectancies: they are positive when experienced value is greater than anticipated, and negative otherwise. Such RPE signals have been identified in midbrain dopaminergic neurons and have also been consistently measured in the *VS*, to which these neurons project (30–32). Hence, dopaminergic projections could facilitate value learning by gating plasticity between sensory information and anticipated value representations in the *VS* and the vmPFC. To establish the causal role of the vmPFC in supporting value learning and anticipation, lesion studies have used different learning paradigms.

The critical role of the vmPFC in learning the rewarding outcome associated with a stimulus was first suggested after seminal work that employed the Iowa gambling task [IGT, Box 1; (33)]. Poor performance in the IGT, however, is not specific to vmPFC damage (34–36). Performance in the IGT is also difficult to interpret, in part because the task involves both deterministic and probabilistic aspects, meaning that stimuli may predict rewarding outcomes either with certainty or a certain degree of probability, as well as apparent reversals in stimulus-value contingencies (37). In tasks involving only deterministic associations between stimuli and reward values, damage to the vmPFC was not found to significantly impair reward learning (38). Tasks involving probabilistic contingencies demonstrated inconsistent impairments and suggested that bilateral vmPFC lesions may be required to reliably affect reward learning (39–42). Furthermore, the vmPFC was shown to be specifically critical for learning the value of stimuli but not for learning the value of actions (39, 43). Instead, successful action-value learning was found to depend on the integrity of the dmPFC, although the vmPFC may be necessary for awareness about action-value relationships (39, 43). In a changing environment, stimulus-value associations can evolve over time, for example when discovering that a previously liked food is toxic, requiring individuals to update the learned value. Deficits in reversal learning tasks have been demonstrated consistently after vmPFC injury (21, 38–40, 42, 44) [but see (41)]. Moreover, reward value can also change as a result of a change in individuals’ internal states, for example when satiated with a particular food. In devaluation tasks, patients with focal damage to the vmPFC demonstrated impaired devaluation, persisting in selecting conditioned stimuli associated with food that had been devaluated through selective satiation (45). Consistent evidence therefore supports the causal role of the vmPFC in the flexible updating of anticipated or learned reward values, which can then be used to guide behavior and decision-making.

BOX 1 **Economic and social games.**

**Table 1:** 

Iowa gambling task	In this task, participants choose cards from four different decks. Each card provides either a gain or a loss. Two decks are “good decks,” providing small gains but also smaller losses, leading to a net gain overall. The two others decks are “bad decks,” providing big gains but even bigger losses, leading to a net loss overall. The goal is to earn as much money as possible.
Dictator game	In this game, one participant decides how to share a monetary amount with an anonymous partner. The recipient only plays a passive role. The amount of money allocated to the partner serves as a measure of the dictator’s deviation from self-interest and provides evidence of the influence of fairness and altruism in social behavior.
Ultimatum game	In this game, a proposer, who is endowed with a sum of money (the stake), must suggest a way to split it with another player, the responder. The responder may accept or reject the offer. If the responder rejects the offer, neither player receives any money. The behaviors of both the proposer and the responder can serve as a measure of fairness preferences.
Trust game	In this game, an investor is endowed with a sum of money and decides how much money to send to a trustee. The amount transferred is then multiplied (e.g., by a factor > 1), and the trustee must decide how much to return to the investor. In single-shot versions of this game, the investor’s behavior is a measure of trust, while the trustee’s behavior is a measure of trustworthiness and social-preferences.
Prisoner’s dilemma game	In this task, two anonymous participants independently decide whether to cooperate or to defect. Each player is paid according to the combination of the two decisions. The payoffs are arranged such that each player will earn the most by defecting, but the team will collectively earn the highest earning if both participants cooperate. Behavior in this task is taken as a measure of social cooperation.
Public good game	In this game, several participants decide how much to contribute to a group pot (i.e., maximizing joints payoffs), which is multiplied and split equally amongst all participants, and how much to keep for themselves (i.e., maximizing individual payoffs by free-riding). Single-shot versions of this game measure social cooperation.
Moral dilemma	In the classic ‘trolley problem’, a trolley is hurtling down a track toward five people who are tied to the rails and cannot move. A lever is within reach that can switch the trolley to a different track where there is only one person tied up. Participants must decide whether to sacrifice one person by pulling the lever in order to save the lives of five others. In the ‘Footbridge’ variation of this dilemma, the person deciding the fate of the individuals must physically push someone off a bridge to stop the trolley and save the others.

### 2.3. Decision values

Decision values signals are thought to measure the difference between the considered option value and another option value, and are used to guide decisions toward the option with the largest benefit. They rely on the net value of options, integrating the anticipated values and costs of each option. Functional imaging studies have identified such value signals at the time of decision in a network of regions, including the vmPFC, during simple binary decisions, for example when choosing between two different food items, drinks, monetary rewards, products, artworks, etc. (12, 13). Decision value signals in the vmPFC have also been shown to integrate the different costs, such as the delay, the effort, or the uncertainty associated with the options of a choice (27, 46–48). The vmPFC has, therefore, been hypothesized to be critical for economic rationality and utility maximization. Damage to the vmPFC has been shown to induce choice inconsistency and choice intransitivity. Choice inconsistency consists in not choosing the same option during repeated choices, or not choosing the option that was given the best rating. Choice intransitivity consists in choosing A over B, B over C, but C over A (39, 49, 50) although not always (51). Yet, when present (but see (51)), these effects are small and vmPFC patients can still make decisions readily. This led to the hypothesis that the vmPFC may not be necessary for rational decision-making *per se*, but rather may promote preference stability by reducing variability in valuation across time (52). In repeated choices, individuals with vmPFC damage express preferences that are indeed more variable, but fundamentally transitive (52). Therefore, vmPFC may not be the only critical structure supporting rational value-based choices, which may rely instead on distributed areas, including the *VS*, that can compensate for damage in the vmPFC (52). Increased choice variability may also participate to explain inconsistent results observed in tasks that assess cost-related preferences. In intertemporal choices that involve choosing between smaller immediate rewards and larger delayed rewards, some studies reported greater temporal discounting of delayed rewards after vmPFC damage (53–57), while others reported no difference compared to controls (58, 59). Similarly, lesions to the vmPFC have been suggested to affect decision-making under uncertainty. Yet, although increased risk-taking has been reported in vmPFC patients (54, 56, 60–62), some studies found no effect (24, 59), one study reported higher risk-taking in the loss-domain but lower risk-taking in the gain-domain (63), and another found increased risk-taking only while receiving dynamic feedback (64). Little data exist on the impact of vmPFC damage in humans on effort- and reward-based decision-making, that involve deciding to make an effort to obtain a reward, but preliminary results suggests that vmPFC may not be critical for such decisions (65). In closely related incentive motivation tasks, that involve effectively producing effort to obtain a reward, damage to the vmPFC reduced the vigor of effort (saccade velocity) produced in response to different reward levels (66). However, here again, the magnitude of this deficit was moderate, in particular in comparison to that observed after lesion to the ventral striato-pallidal complex which completely abolishes effort modulation (67, 68). This series of lesion studies therefore suggest that, at the time of decision or action, the vmPFC may not be critical, but may play a modulating role in shaping value-based behavior. Interestingly, recent findings on valuation about multidimensional options could shed light on the specific role of the vmPFC in decision-making. When value has to be inferred from the multiple attributes of a stimulus, vmPFC patients differ from prefrontal lesioned and healthy control individuals in how they weight the different attributes in certain choices, for example when choosing artworks based on their perceptual, conceptual and affective characteristics (69), but not in other, for example when choosing potential houses based on their features (70). Recent work suggested that vmPFC damage might specifically affect decision-making when value must be inferred from the unique combination of attributes, in other words their interaction, and not when value can be inferred from the sum of independent attributes (71). Such a role could potentially account for why vmPFC patients are not incapable of making choices, but systematically deviate from healthy individuals in their decisions.

## 3. Social values in the vmPFC

Beyond its role in economic valuation, the vmPFC has been implicated early in shaping social behavior. In the 1970s and 1980s, the terms “pseudopsychopathy” (72) and “acquired sociopathy” (2) were coined to describe the dramatic personality and behavioral changes in the social realm observed in patients with vmPFC damage: blunted affect, lack of empathy, poor tolerance to frustration and irritability, social inappropriateness and antisocial behaviors (73–78). However, the precise cognitive mechanisms underlying these social deficits have remained elusive. In the following sections, we review the lesion studies that have examined the role of the vmPFC in supporting social value signals and which may support the “extended common neural currency” model. To simplify the wide range of contexts and decision types encountered in these studies, we group them into three classes of social values: the value that one assigns to other individuals, the value of outcomes that benefit others, and the value of outcomes that conform to social norms (11).

### 3.1. Value assigned to others

Most people tend to pursue social interactions that offer some form of gratification or benefit. The first class of social valuation therefore concerns situations in which individuals assess the personal value of another person, for example when judging the attractiveness or trustworthiness of a person, or situations in which they assess the value of another person’s actions to themselves, for example when being applauded by someone or when having trust reciprocated by someone. Functional imaging studies have found neural activation in the vmPFC when receiving, or anticipating rewarding social outcomes, such as viewing faces with positive affects (79), attractive faces (80), erotic photos (81), or when receiving social approval or romantic interest from others (82–84). Neural activity in the vmPFC also predicts subsequent decisions about liked others, such as the willingness to pay to view attractive faces (85) or to donate to preferred charities (86). BOLD activity in the vmPFC is also associated with learning about moral values of others, such as their honesty and trustworthiness (87, 88) and with the willingness to reciprocate trust (89). Consistent with imaging studies, lesion studies support the causal involvement of the vmPFC in the valuation of others. First, vmPFC damage reduces the tendency to approach positive and avoid negative emotional faces, particularly for negative affect, while preserving the ability to recognize facial expressions (90–92). Similarly, patients with vmPFC lesions have lower inter-personal disgust, showing less reluctance to interact with unsavory others or with individuals described as socially deviant (93). They also show lower consistency when choosing between potential spouses, based on non-physical attributes (94). Additionally, vmPFC damage affects social judgment about others, decreasing for example the perceived trustworthiness of unknown individuals (95, 96), and impairing the ability to revise these judgments based on the individuals’ observed social and moral conducts (97). These findings demonstrate consistent impairment in behaving according to the value attached to other individuals after damage to the vmPFC.

### 3.2. Value of outcomes benefiting others

People are not always driven by their own self-interest and frequently take into account the well-being of others. Thus, a second class of social value signals correspond to the vicarious valuation of outcomes that are rewarding for others, adopting their perspective, for example when rejoicing in someone’s victory, or when choosing to cook for someone his or her favorite food. Empirical studies have found that experiencing reward when directly receiving positive outcomes, or when observing others receiving such outcomes, activated the same regions of the brain valuation system, in particular the vmPFC (98). Experiencing reward for others also consistently recruit the *VS* (99, 100), although this structure may be preferentially activated in response to personal as compared to vicarious reward (98). Importantly, vicarious value signals in the vmPFC have been shown to predict choices during decisions that result in benefits for others, such as purchasing DVD movies or selecting monetary rewards for others (101–103). Additionally, the vmPFC encodes reward prediction error signals that support learning about another person’s preferences (104, 105). By contrast, bilateral damage to the vmPFC reduces empathic tendencies toward others in clinical questionnaires (76). In economic games, such as the dictator and the ultimatum game (Box 1), patients with focal vmPFC lesions showed impaired concern about payoff to others, giving less money to anonymous strangers (95), even to individuals who are suffering (106). In the trust game (Box 1), when they are endowed from an investor with a sum of money that is later tripled, patients with vmPFC damage make lower back transfer to the investor than healthy controls (96). Taken together, these results therefore provide support for a critical role of the vmPFC in other-regarding preferences. *Empathy*, the ability to share another person’s feelings, has been conceived as an initial step that can motivate such other-regarding motivation, or *sympathy,* and is considered one of the fundamental motives driving altruistic acts, which entail personal costs for the benefit of others (107).

### 3.3. Value of social norms and principles

People’s behavior is not solely determined by their own interest or by the interest of specific others, but is also shaped by the collective welfare. A third class of social value signals therefore consists in the valuation of options or outcomes according to their conformity with normative social principles, for example when rejoicing in a fair distribution, or when turning down a bribe. Functional imaging studies have consistently reported neural value signals in reward-related brain regions in relation to social principles, such as fairness, cooperation or morality. For example, in economic exchange tasks such as the ultimatum game (Box 1), a fair distribution of money among players is perceived as rewarding and is associated with neural activation in the ventral striatum and vmPFC (108, 109). By contrast, inequality has been associated with activation of neural networks involved in conflict and aversive outcomes, including dorsal ACC and anterior insula (110). When given the opportunity, people tend to punish norm violators who propose unfair distributions, even when this is costly for them (111). Punishing defectors is thought to promote social norms enforcement (112), and is perceived as rewarding and elicits activation in the vmPFC (111). Another example of social principle eliciting neural value signals is cooperation. Research using the Prisoner’s Dilemma game (Box 1) has shown that mutual cooperation is associated with increased activation in reward-related brain regions, including the vmPFC (113). These results have been proposed as evidence of the intrinsic value of cooperation, which can motivate individuals to engage in prosocial behavior and collective action. Finally, value signals have also been reported in the vmPFC during moral dilemmas (Box 1), for example when judging the moral acceptability of sacrificing a single life to save a larger group of dying (114). These findings suggest that normative social principles have inherent values that are encoded in the vmPFC. Damage to this region may therefore impact on how such principles shape human behavior. Lesion studies have, however, provided conflicting results on the antisocial or prosocial effects of vmPFC damage. In the ultimatum game (Box 1), vmPFC patients were initially reported to reject unfair offers at a higher rate than healthy controls, although showing normal levels of anger following unfair offers (95, 115, 116). These patients would also demand, as responders, the same amount that they offered as proposers, whereas controls generally offer more than they demand (95). This result has been interpreted as reflecting an insensitivity to guilt in vmPFC patients, defined as the aversion for advantageous inequity (95). This finding was replicated in another study, but only when monetary gains were presented as abstract amounts to be received later (117). When offers were readily available in cash, vmPFC patients showed normal rejection rate of unfair offers. Inconsistently, a recent study found that vmPFC patients showed diminished sensitivity to unfairness and were more willing to accept unfair offers than control participants (118). Research on the impact of vmPFC damage on cooperation is more limited, but one study reported that vmPFC patients were more likely to cooperate in a public good game (Box 1), with the opposite being true for dlPFC patients who cooperated less than control patients (94). These preliminary results challenge the view that vmPFC is a necessary component for cooperative behavior. By contrast, the impact of vmPFC damage on moral judgments is well-established. In moral dilemmas that involve causing the death of one person to save several lives, patients with vmPFC damage are more likely to choose the utilitarian option, that consists in sacrificing one person, than control individuals (116, 119–122). Although early works suggested that vmPFC patients might be especially impaired in personal versus impersonal moral dilemmas, when directly versus indirectly causing harm, further work showed that they endorsed utilitarian actions more often than healthy individuals, regardless of the situation (122). This utilitarian behavior has been interpreted as reflecting a lack of automatic affective response to moral transgressions in vmPFC patients, which was supported by the absence of autonomic skin response prior to such violations (120). Consistently, when judging the morality of actions made by others, vmPFC patients relied more on the outcome of the actions (i.e., whether they were harmful or not) than on the intention with which the actions were pursued (i.e., whether they were intentional or accidental), suggesting that vmPFC is necessary for integrating both intention and outcome into a moral value (123, 124). Although the impact of damage to the vmPFC on decision-making based on social principles, such as fairness, cooperation and morality, is often reported, it is not yet fully understood why such damage can result in both prosocial and antisocial behaviors.

## 4. Discussion and open questions

Most of the findings discussed above support the notion that the vmPFC represents neural value signals both in social and non-social contexts, consistent with the “extended common neural currency” hypothesis. However, the extent to which the vmPFC is critical for value processing may depend on the specific stage of the decision-making process or the nature of the rewarding outcome. In the economic domain, while the vmPFC may not be strictly necessary for experiencing the hedonic value of rewards, its integrity seems essential for learning and flexibly updating reward value. By contrast, empirical studies have produced mixed results regarding the effects of vmPFC damage on choices: some studies demonstrated deficits or biases that were moderate or restricted to certain types of decisions, while others showed preserved abilities to make consistent choices. In the social domain, lesion studies support the critical role of the vmPFC in assigning a subjective value to other individuals. They also support its necessary role in vicariously representing the benefits obtained by others. Damage to the vmPFC also demonstrated its critical role in moral judgments but showed inconsistent deficits in decision-making based on other normative social principles such as cooperation and fairness. The inconsistent effects of vmPFC’s lesions on choices in certain social and non-social contexts suggest that the vmPFC may exert a modulatory rather than a necessary role at the time of decision. Alternatively, it is possible that the vmPFC is involved in certain types of valuation more specifically, for example when an option’s value can only be determined by the interaction between its attributes and not by attributes independently (71).

The common involvement of the vmPFC in both social and non-social contexts raises the question of whether valuation in these contexts relies on the same neurons, or on distinct sub-regions or populations of neurons within the vmPFC. Meta-analyses have suggested a possible posterior-to-anterior gradient of value representations corresponding to concrete-to-abstract rewards (13). Yet, only few imaging studies have directly compared social and non-social value signals using the same experimental design (81, 104, 125–127). One of these studies observed a ventral-to-dorsal gradient in the processing of self- vs. other-regarding value signals (127). While lesion studies do not provide the necessary anatomical specificity to dissociate the roles of distinct areas within the vmPFC, single-unit recordings studies have started to identified neurons that may selectively encode social vs. non-social aspects of rewards both in non-human primates (128, 129) and in humans (130). However, it is worth noting that a clear distinction between social and non-social decisions can be subject to debate. Many decisions in people’s lives, which may not involve another person at first glance, may, in fact, carry a social component. Consider, for example, the strong social influence that can impact the value people assign to material goods, such as when they decide to eat a vegetarian meal or buy a luxury car. Further works will help clarify whether the valuation of stimuli associated with social versus non-social contexts is implemented by common or overlapping but specialized neuronal populations.

**Figure 2:**
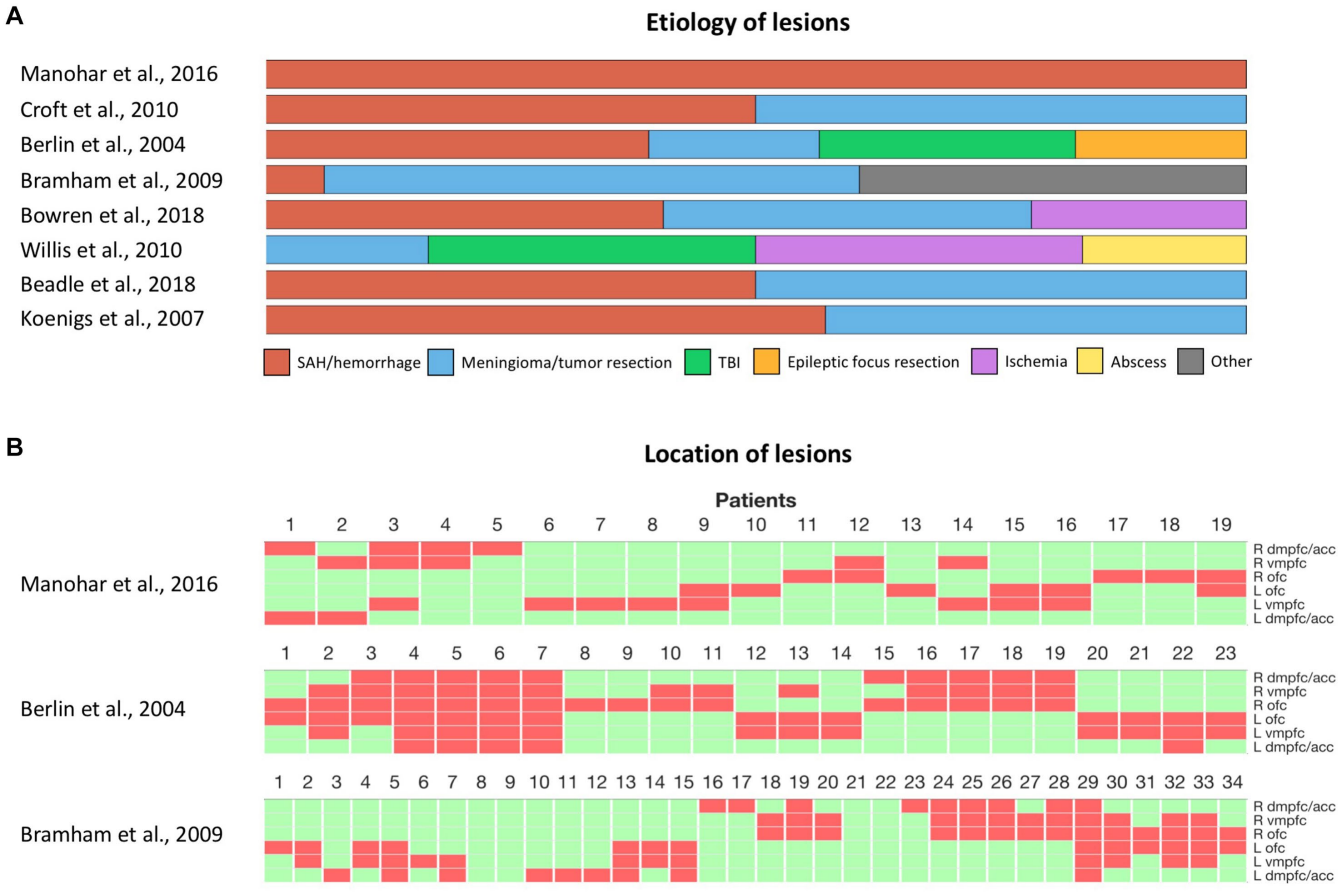
Heterogeneity of vmPFC lesions in human studies. **(A)** Etiology of vmPFC lesions among cited studies with individual lesion data. **(B)** Location of lesions among cited studies with individual lesion data. Each column represents a patient. Green and red rectangles represent preserved and damaged areas, respectively. Brain areas are represented from top to bottom in the following order: right dmPFC/ACC, right vmPFC, right OFC, left OFC, left vmPFC, left dmPFC/ACC. SAH, subarachnoid hemorrhage. TBI, Traumatic brain injury. dmPFC, dorsomedial prefrontal cortex. ACC, anterior cingulate cortex. vmPFC, ventromedial prefrontal cortex. OFC, orbitofrontal cortex.

The inconsistent effects observed after damage to the vmPFC, both in social and non-social decision-making, also question the influence of the heterogeneity of lesions across patients (Figure 2). A first factor that may contribute to these inconsistencies is the variability in the nature of the lesions across different studies (Figure 2). Ischemic, hemorrhagic, traumatic, or surgical causes can lead to different distributions of lesions and varying degrees of functional impairment. Samples consisting primarily of orbital meningioma cases involve more bilateral and anterior lesions, while vascular lesions often concern more posterior and unilateral lesions (14). Moreover, although surgical resection of brain tumors can lead to a greater degree of functional impairment compared to vascular lesions or traumatic brain injuries, the slow progression of tissue damage, as the tumor expands, may allow for brain plasticity and functional compensation over time (131). The age of lesion onset may also significantly influence the degree of functional impairment. Early-onset lesions that impact neurodevelopment tend to lead to more pronounced deficits (132–134). The age of lesion onset may also interact with changes in brain plasticity across the lifespan, and thus condition functional recovery. Thus, an important guideline for future studies would be to report the nature and the location of individual lesions, as well as the age of lesion onset when describing vmPFC patients (14). Finally, the variability in the impact of vmPFC damage raises the question of possible hemispheric specialization or functional redundancy between left and right vmPFC. Bilateral lesions in the vmPFC are rare and most of the patients included in the lesion studies mentioned above had unilateral lesions (Figure 2). Thus, it remains possible that compensation from the preserved vmPFC masked the effects of unilateral vmPFC damage. However, the limited sample size of vmPFC studies, which classically include only a tenth of patients or fewer, hinder the ability to compare the effects of bilateral and unilateral damage to the right or left vmPFC. Small samples also limit our ability to investigate deficits that arise from the interaction of lesions in the vmPFC and another region. Therefore, future research should aim to include larger samples of patients, possibly through multicenter collaboration, with a sufficient number of bilateral lesions, to investigate the potential effects of laterality, redundancy, and interactions following vmPFC damage.

Another question that remains is how specific the role of the vmPFC is in social and non-social decision-making, as such complex behaviors are likely to involve a network of multiple brain regions. While lesion studies shed light on the contribution of the vmPFC, few of them directly compared the effects of damage to the vmPFC to damage in other parts of the prefrontal cortex. Therefore, including control patients with prefrontal lesions outside the vmPFC in future studies would be essential to gaining a more comprehensive understanding of the unique role of the vmPFC in decision-making. Moreover, there is also evidence that the vmPFC receives specialized inputs from specific brain regions when constructing values in social and non-social contexts. For example, structures outside the classical reward circuitry and typically associated with social cognition, such as the dmPFC, dlPFC and TPJ, have been shown to be preferentially engaged in response to vicarious as compared to personal reward (98), and are thought to provide information that are relevant for the construction of social values through their connectivity with the vmPFC (11, 86). Further research is needed to provide a more comprehensive understanding of how remote cortical areas provide inputs for the computation of values in both social and non-social contexts. Additionally, investigating the impact of lesions in these structures and in the subcortical and white matter pathways that convey specific information to the vmPFC could shed light on diverse biases in decision-making.

In this review, we hope to have summarized the evidence supporting the notion that the vmPFC encodes value signals at different stages of the decision-making process, when receiving, learning and deciding about valued outcomes, both in the economic domain and in various social contexts, for example when valuating other individuals, others’ benefit, or social normative principles. In the modern era, non-invasive brain stimulation techniques, such as Transcranial Magnetic Simulation (TMS) and Transcranial Direct Current Stimulation (tdCS), are classically used to demonstrate causality in brain functions. Yet, these methods can hardly modulate neural activity in subcortical structures or medial cortical areas as they primarily target brain areas at the cortical surface. Here, we have therefore also tried to emphasize the invaluable contribution of lesion studies in establishing or challenging causal brain-behavior relationships, particularly in reward-related brain areas, like the vmPFC or the ventral striatum. Overall, neuroimaging and lesion studies support an “extended common neural currency” schema, where the vmPFC serves as key motivational node that shapes both social and non-social human behaviors, but flexibly integrates inputs specific to the decision-making context.

## Author contributions

RB wrote the first draft of the manuscript. RB and DM wrote sections of the manuscript. All authors contributed to manuscript revision, read, and approved the submitted version.
